# Comparative analysis using machine learning methods for determination of pharmaceutical solubility in supercritical CO_2_ considering physical properties of drugs

**DOI:** 10.3389/fmed.2026.1921513

**Published:** 2026-09-03

**Authors:** Saad M. Alshahrani

**Affiliations:** Department of Pharmaceutics, College of Pharmacy, Prince Sattam Bin Abdulaziz University, Al-Kharj, Saudi Arabia

**Keywords:** Histogram-Based Gradient Boosting, machine learning, Neural Additive Models, pharmaceutical solubility, supercritical fluid

## Abstract

**Background:**

Reliable prediction of pharmaceutical solubility in supercritical carbon dioxide (SC-CO_2_) can support particle engineering and formulation design. However, record-wise validation may overestimate generalization when measurements for the same compound occur in both the training and test sets.

**Methods:**

A leakage-aware machine-learning workflow was evaluated using molecular weight, melting point, temperature, and pressure as predictors of drug solubility in SC-CO_2_. Experimental records compiled from published measurements were screened using Isolation Forest and scaled by min-max normalization. Kernel ridge regression (KRR), histogram-based gradient boosting (HGB), and neural additive models (NAMs), together with linear regression and multilayer perceptron baselines, were evaluated using a 90/10 record-wise split and leave-one-compound-out (LOCO) validation. Hyperparameters were tuned using Hyperband.

**Results:**

HGB provided the strongest overall performance. Under the random record-wise test split, it achieved R² = 0.99372, RMSE = 0.247192 g/L, and MAE = 0.113038 g/L. Under the more stringent LOCO evaluation, its performance decreased to R² = 0.93128, RMSE = 0.498217 g/L, and MAE = 0.271894 g/L. The difference between the two validation schemes demonstrates that random splitting yields an optimistic estimate when compound-specific observations are shared across subsets.

**Conclusion:**

HGB accurately correlated solubility within the investigated data distribution and retained useful predictive ability for compounds excluded from model training. Nevertheless, external validation and additional molecular descriptors are required before extrapolating the model to chemically distinct pharmaceuticals.

## 1 Introduction

Limited aqueous solubility remains a critical obstacle in developing effective solid pharmaceutical dosage forms because it can restrict drug dissolution, bioavailability, and therapeutic performance. Many active pharmaceutical ingredients exhibit inherently low water solubility, prompting the development of various formulation strategies. One widely investigated approach is the conversion of crystalline drugs into higher-energy amorphous forms, particularly through amorphous solid dispersions (1–3). Advances in supercritical-fluid technologies have also introduced versatile particle-engineering routes for pharmaceutical manufacturing. These processes can generate micro- and nanoscale drug particles with increased surface area, thereby promoting faster dissolution and improved apparent solubility in aqueous environments. Consequently, several supercritical-fluid-based techniques have been developed for producing pharmaceutical nanoparticles, with drug solubility in the supercritical medium serving as a fundamental parameter for process design and operating-condition selection (4, 5).

Various theoretical modeling strategies have been introduced to quantify pharmaceutical solubility in supercritical solvents, with thermodynamic approaches remaining the principal framework for estimating drug solubility under supercritical-fluid conditions. Activity coefficient-based models as well as equation of state models are among the most widely used thermodynamic methods, which have been utilized in correlation of solubility values in supercritical CO_2_ (6–10). Their principle is based on phase equilibria for solid and liquid (solvent) by computation of unknown parameters using the measured data. Considering a vast range of medications that should be nanosized using the supercritical method, the development of thermodynamic models for each drug is challenging and can fail to meet the requirements. Thus, the models of learning nature are preferred, such as machine learning, due to their capacity to handle large datasets and offer unique properties in the estimation of solubility.

The capacity of machine learning (ML) algorithms to identify intricate and nonlinear patterns in scientific datasets has substantially expanded their application across chemistry, biology, and materials science. In the field of supercritical-fluid technology, numerous data-driven models have consequently been investigated for predicting pharmaceutical solubility. For example, Bahrami et al. (11) employed an adaptive neuro-fuzzy inference system (ANFIS) to estimate drug solubility in supercritical CO_2_ and reported strong predictive performance. The suitability of ML for this application has been further demonstrated by several regression-based studies in which models trained on experimental measurements successfully reproduced pharmaceutical solubility behavior (12–16).

Machine-learning algorithms derive predictive relationships directly from observed data, allowing them to represent complex nonlinear dependencies without requiring a predetermined mechanistic equation. This capability is particularly valuable for estimating pharmaceutical solubility in supercritical CO_2_, where conventional thermodynamic calculations may involve substantial mathematical complexity and compound-specific parameterization. Accurate solubility prediction is essential in pharmaceutical engineering because this property affects formulation design, particle production, material processing, and operating-condition optimization, while being simultaneously governed by molecular characteristics and process variables. The present work therefore applies a structured ML workflow—including data preprocessing, model training, and hyperparameter tuning—to a multi-compound dataset containing molecular weight, melting point, temperature, and pressure as predictors. By considering chemically diverse pharmaceuticals within a unified modeling framework, the study seeks to overcome the restricted applicability of many previously reported models developed for individual compounds and to improve predictive generalization across a broader chemical domain.

To examine the trade-offs among predictive accuracy, computational efficiency, and interpretability, three methodologically distinct algorithms were benchmarked: Kernel Ridge Regression (KRR), Histogram-Based Gradient Boosting (HGB), and Neural Additive Models (NAMs). Although related techniques—especially boosting-based algorithms—have been used in QSPR and QSAR research, comparative assessments of these three approaches on heterogeneous pharmaceutical datasets spanning multiple compounds and thermodynamic conditions remain scarce. KRR uses the kernel trick to represent nonlinear patterns in an implicitly transformed feature space, making it appropriate for systems governed by complex variable dependencies. HGB constructs boosted decision trees from discretized feature values, thereby reducing computational demand while retaining strong performance on structured tabular data. In contrast, NAMs parameterize the contribution of each predictor through an individual neural subnetwork, combining the nonlinear learning capacity of neural networks with the feature-level transparency of additive models. This interpretability facilitates identification of the molecular and operating factors that govern solubility behavior.

This work makes three principal contributions. The first is the establishment of a structured machine-learning workflow specifically designed for solubility estimation, in which Isolation Forest-based anomaly screening and min–max feature scaling are incorporated to obtain a consistent and reliable dataset for model development. Second, it conducts a comparative analysis of KRR, HGB, and NAMs, highlighting their ability to model complex solubility relationships while balancing accuracy and interpretability. Third, it provides detailed visualizations, including single-feature effect plots and contour plots, to elucidate the influence of individual and paired input features on solubility, offering valuable insights for chemical modeling applications. These contributions advance the application of ML in chemical engineering, paving the way for scalable and interpretable predictive models.

Although the present study uses the experimental compilation reported in Ref. (11), its contribution does not rest on the construction of a new database. Whereas Ref. (11) evaluated GEP and ANFIS models using random record-level data partitions, the present work compares three complementary modeling paradigms—kernel-based regression, histogram-based tree boosting, and an intrinsically interpretable additive neural architecture—and further benchmarks the best-performing model against linear regression and a standard multilayer perceptron. More importantly, leave-one-compound-out evaluation is employed to assess transferability to pharmaceutical compounds entirely excluded from model training, thereby distinguishing compound-level generalization from interpolation among repeated measurements of previously observed drugs. Residual diagnostics, applicability-domain assessment, and single-feature and ALE analyses further extend the evaluation from predictive accuracy to error structure, prediction reliability, and interpretable physicochemical behavior.

## 2 Methodology

A structured data-driven methodology is adopted to estimate pharmaceutical solubility by capturing the complex dependencies between molecular characteristics and operating conditions through advanced ML algorithms. Model development is based on the dataset reported in Ref. (11), which comprises experimentally measured solubility values for multiple pharmaceutical compounds in supercritical CO_2_ over a range of thermodynamic conditions. It should be noted that this dataset represents an aggregated collection derived from previously published experimental studies rather than a newly curated multi-source dataset. While it includes a diverse set of compounds and operating conditions, the exact distribution across drug classes and experimental protocols may not be fully uniform. Consequently, there is a possibility of underlying biases or systematic variations inherited from the original experimental sources, which should be considered when interpreting the model outcomes.

**Figure 1:**
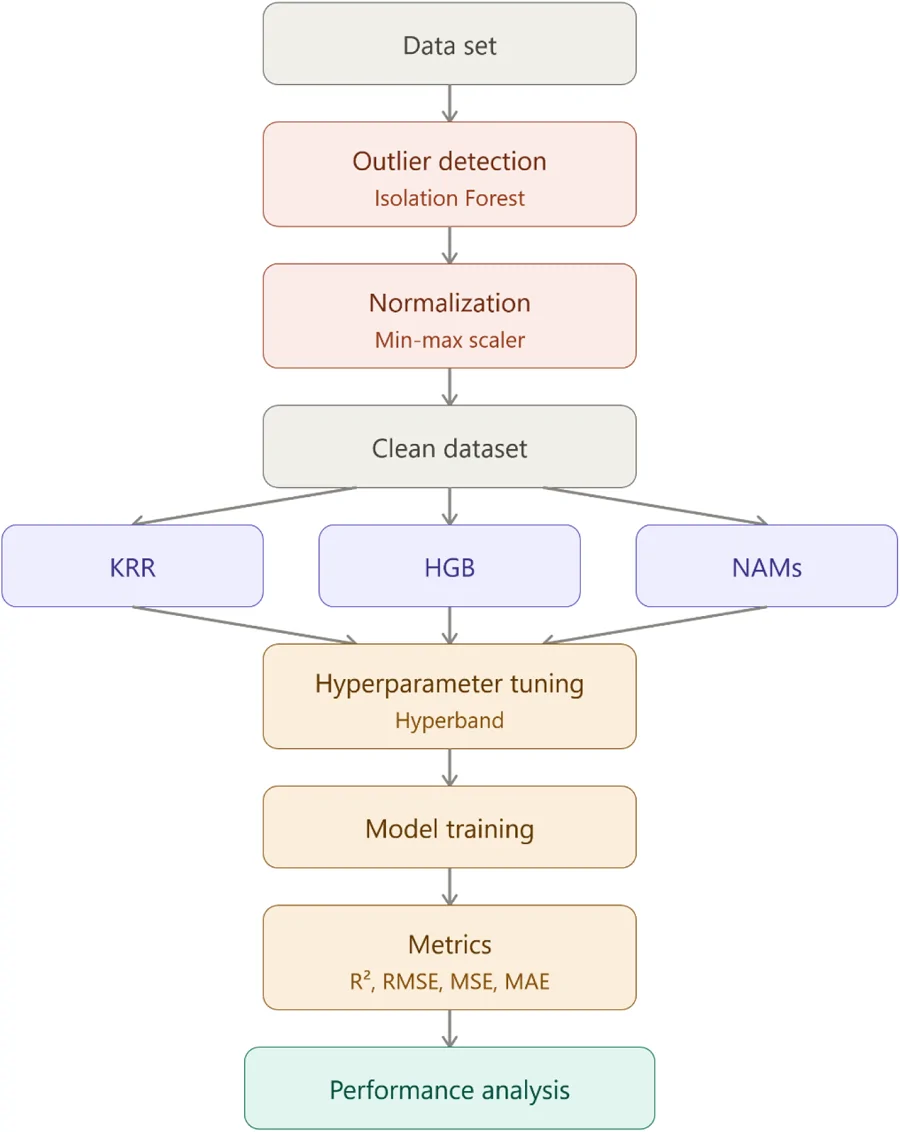
Flowchart of the machine learning pipeline.

The methodology is structured into several key phases including preprocessing of raw data, model development, hyperparameter optimization, and performance assessment for all models. Initially, the dataset undergoes rigorous preprocessing, including outlier detection using Isolation Forest and feature normalization via min-max scaling, to ensure the data is clean and suitable for modeling. Three distinct machine learning models—KRR, HGB, and NAMs—are then employed to capture both linear and nonlinear relationships within the data. Hyperparameter tuning is conducted using Hyperband, an efficient optimization algorithm that balances exploration and exploitation to identify optimal model configurations. Finally, the models are evaluated using metrics such as R^2^, RMSE, MSE, and MAE on training, validation, and test sets to assess their predictive accuracy and generalizability. This comprehensive methodology ensures a robust and interpretable framework for solubility prediction, with a focus on both performance and transparency. Figure 1 displays the flowchart of the ML pipeline designed for the correlation of drug solubility to the input features and validation of models.

Compound identity was used as the grouping variable for leave-one-compound-out evaluation. The dataset contained 66 unique compounds, resulting in 66 LOCO folds. In each fold, all measurements belonging to one compound were excluded from training and used exclusively for testing. Isolation Forest and min-max scaling were refitted using only the training compounds in that fold and then applied to the held-out compound. Predictions from all folds were concatenated, and R^2^, RMSE, MSE, and MAE were calculated once from the pooled out-of-fold predictions. Hyperparameter selection was performed independently within each outer LOCO training fold using Hyperband with five-fold grouped cross-validation, where compound identity was retained as the grouping variable. Thus, the compound held out in each outer fold remained completely unseen during preprocessing, hyperparameter optimization, and model fitting.

### 2.1 Data preparation

**Figure 2:**
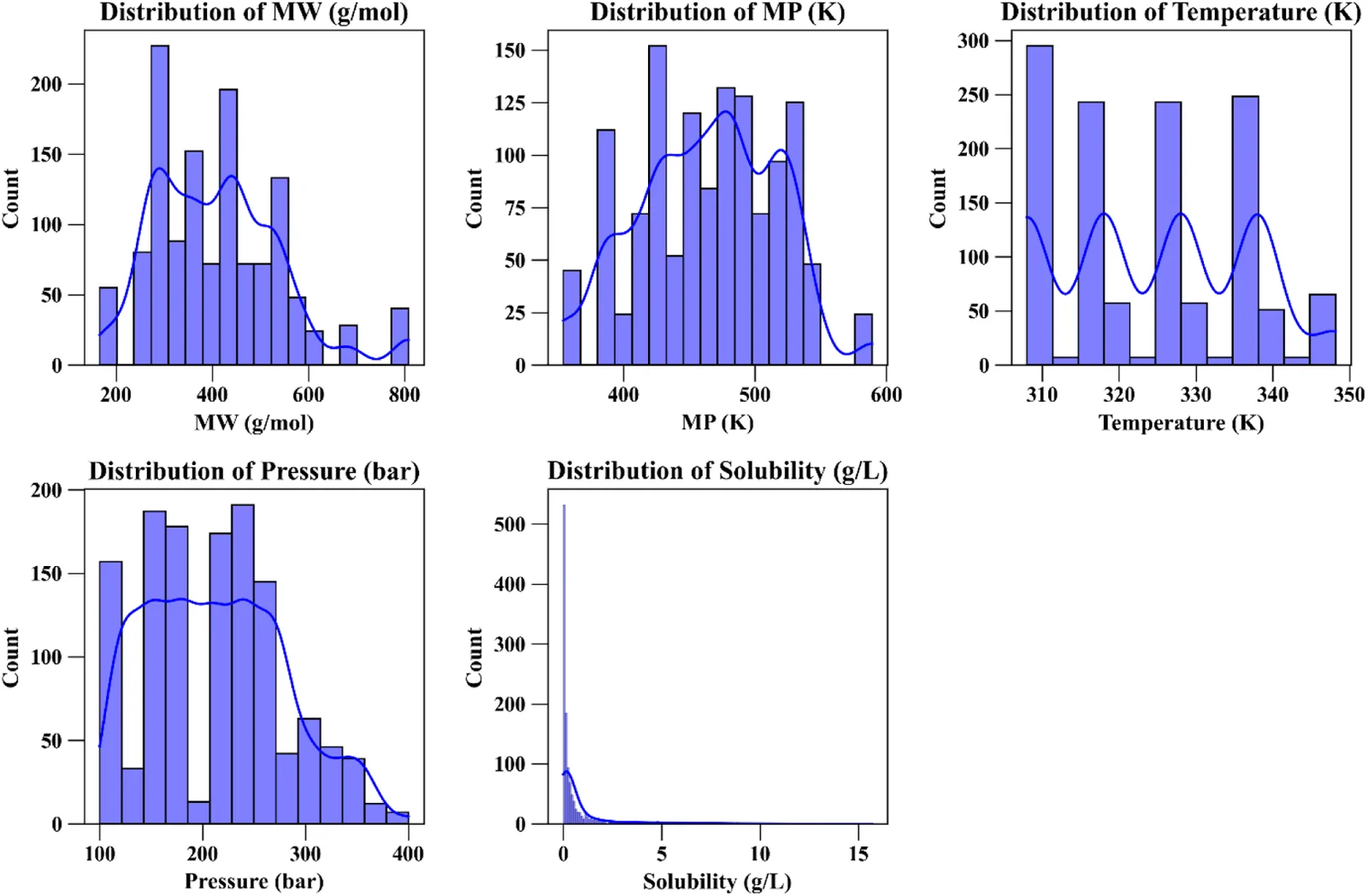
Histograms of variables in the dataset, including drug's molecular weight and melting point, as well as process parameters, including pressure and temperature. The sole response is drug solubility (g/L).

This study utilizes a comprehensive dataset of over 1,600 solubility measurements for diverse chemical compounds, each recorded under varying temperature and pressure conditions. Key variables include molecular weight (g/mol), melting point (K), measurement temperature (K), pressure (bar), and solubility (g/L). This rich dataset enables a thorough analysis of how molecular and environmental factors influence solubility, supporting the development of predictive models with strong generalizability (11). Histograms of input and output variables are shown in Figure 2.

The dataset covers a temperature range of approximately 308–338 K and a pressure range of approximately 8–40 MPa, representing typical operating conditions for solubility measurements in supercritical CO_2_ systems. These ranges define the primary domain within which the developed models are expected to provide reliable predictions. Predictions outside these ranges should be interpreted with caution, as the model is not trained for extrapolation beyond the observed experimental domain. Data preparation is an important stage in ML pipelines since it ensures that the incoming data is clean, consistent, and ready for modeling. In this study, outlier detection was performed using the Isolation Forest algorithm to identify and handle anomalous solubility measurements within the dataset of over 1,600 chemical compounds. Isolation Forest (17, 18) is an ensemble-based anomaly detection method built from a collection of randomized binary trees, known as isolation trees. Each tree is constructed by recursively and randomly selecting a feature—such as molecular weight, melting point, temperature, pressure, or solubility—and a random split value within that feature's range, thereby partitioning the data into progressively smaller subsets. Because anomalous points tend to be few and distinguishable from the bulk of the data, they are isolated into their own partition after only a small number of splits, resulting in a short average path length from the root of the tree. In contrast, normal points, which lie in denser regions of the feature space, require substantially more splits to be isolated, resulting in longer average path lengths. Averaging the path length for each observation across all trees in the ensemble yields an anomaly score bounded between 0 and 1: scores close to 1 indicate a high likelihood of being an outlier, while scores close to 0.5 or below indicate normal behavior. Observations are then flagged as anomalies if their score exceeds a threshold set by the contamination parameter, which specifies the expected proportion of outliers in the dataset; in this study, the model was configured with the default 100 estimators (trees) and the default contamination setting (“auto”), which lets the algorithm determine the anomaly threshold based on the original Isolation Forest scoring function rather than a user-specified outlier proportion. This method is particularly effective for high-dimensional datasets like the one used here, as it identifies anomalies based on how easily a point can be isolated rather than relying on distance measures or density estimation, making it robust to the scale of the data and eliminating the need for prior normalization. Its computational efficiency and scalability further make it an ideal choice for preparing this dataset for subsequent modeling. Based on this configuration, no data points were identified as anomalous. Therefore, no samples were removed or altered, and the entire dataset was retained for model development. This outcome suggests that the dataset is internally consistent and does not contain extreme deviations that would adversely affect model training. Moreover, retaining all observations ensures that potentially meaningful but rare physicochemical behaviors are preserved in the analysis.

Following outlier detection, data normalization was applied using the min-max scaler to rescale the features to a common range of [0, 1]. The min-max scaler transforms each feature—such as molecular weight (g/mol), melting point (K), measurement temperature (K), pressure (bar), and solubility (g/L)—based on its minimum and maximum values, preserving the relationships between data points while ensuring all features contribute equally to downstream models (19). Although Isolation Forest itself does not require normalization due to its tree-based structure, which is invariant to monotonic transformations, normalization was employed as part of the broader preprocessing pipeline. This step ensures that the data is consistently prepared for models like KRR, HGB, and NAMs, which may benefit from normalized inputs to improve performance and convergence. The min-max scaler's simplicity and ability to handle datasets with outliers, without assuming a specific distribution, make it a suitable choice for enhancing the robustness and generalizability of the predictive models in this study.

The complete dataset was partitioned into 90% training records and 10% test records before initiating any preprocessing procedure. Isolation Forest was calibrated solely with the training observations for anomaly screening, while the minimum and maximum values required for feature scaling were likewise derived only from this subset. The resulting preprocessing rules were subsequently applied unchanged to the held-out test data. Maintaining this separation prevented the test observations from influencing data preparation, hyperparameter estimation, or model fitting, thereby reducing information leakage and supporting an unbiased and reproducible performance assessment. The mathematical formulations and objective functions used for model development are presented in Equations 1–5.

### 2.2 Kernel ridge regression

Kernel Ridge Regression (KRR) is a non-parametric supervised algorithm that extends ridge regression through kernel-based learning to capture nonlinear dependencies within a dataset. Rather than imposing a direct linear association between the predictors and response, KRR uses the kernel trick to represent observations implicitly in a higher-dimensional feature space, where complex input–output patterns can be approximated more effectively by linear functions (20).

Given a training set $[\left(x_{i},y_{i}\right){]}_{i=1}^{n}$, with $x_{i}\in R^{d}$ and $y_{i}\in R$, the KRR model seeks a function *f* from a Reproducing Kernel Hilbert Space (RKHS) that minimizes the regularized empirical risk (21, 22):

$$L(f)=\overset{n}{\underset{i=1}{\sum }}\;(y_{i}-f(x_{i}){)}^{2}+\lambda ∥f{∥}_{H}^{2}$$ (1)

where $∥f{∥}_{H}$ denotes the norm in the RKHS associated with a positive-definite kernel k(⋅,⋅), and λ>0 is a regularization parameter that controls the trade-off between data fidelity and model complexity.

An RKHS H is a Hilbert space of functions $f:R^{d}\to R$ uniquely associated with a positive-definite kernel *k*, satisfying the reproducing property $f(x)={\left\langle \;f\;,k(x,\cdot )\right\rangle }_{H}$ for every f∈H and every *x* in the input domain; in particular, evaluating *f* at a point *x* is equivalent to taking its inner product with the kernel function centered at *x*. This property guarantees that function evaluation is a continuous, well-behaved operation and that the space is fully characterized by the kernel itself. For a function of the form $(\cdot )=\sum_{i=1}^{n}\alpha_{i}\;k(\cdot ,x_{i})$, the RKHS norm reduces to $∥f{∥}_{H}^{2}=\sum_{i=1}^{n}\sum_{\;j=1}^{n}\alpha_{i}\alpha_{j}\;k(x_{i},x_{j})=\alpha^{⊤}K\alpha$, where *K* is the Gram matrix defined below. This norm penalizes functions that are highly oscillatory or complex relative to the geometry induced by the kernel, so minimizing $∥f{∥}_{H}^{2}$ alongside the data-fit term favors smoother, less complex functions and helps prevent overfitting.

By the Representer Theorem (23), the minimizer $f^{*}$ of this problem can be expressed as a weighted sum of kernel functions evaluated at the training points:

$$f^{*}(x)=\overset{n}{\underset{i=1}{\sum }}\;\alpha_{i}\;k(x,x_{i})$$ (2)

where $\alpha =(K+\lambda I{)}^{-1}y$, $K\in R^{n\times n}$ is the Gram matrix with entries $K_{ij}=k(x_{i},x_{j})$, and *I* is the identity matrix. The choice of kernel—such as the radial basis function (RBF), polynomial, or Laplacian kernel—determines the hypothesis space and directly influences the model's ability to capture nonlinear structure. In this study, the RBF kernel, $k(x,x^{\prime })=\exp\;(-\gamma ∥x-x^{\prime }{∥}^{2})$, was used (Table 1), which corresponds to an infinite-dimensional RKHS whose functions are infinitely smooth; this choice enables KRR to approximate highly nonlinear solubility–property relationships without prescribing an explicit functional form.

**Table 1:** Optimal hyperparameters of final models.

Model	Hyperparameter	Value
KRR	Kernel	RBF
	Regularization (*λ*)	0.01
	Gamma	0.1
HGB	Number of trees (n_estimators)	200
	Learning rate	0.05
	Max depth	6
	Max bins	255
	Min samples leaf	20
NAMs	Hidden layers (per feature network)	2
	Neurons per layer	64
	Activation	ReLU
	Learning rate	0.001
	Epochs	100
	Batch size	32

KRR is particularly effective for small to medium-sized datasets, where exact kernel matrix inversion is computationally feasible. It offers closed-form solutions, smooth function estimates, and principled regularization, making it a strong candidate for regression tasks requiring nonlinear modeling with controlled complexity. Additionally, its dual formulation enables natural extension to multi-output regression and structured prediction tasks.

### 2.3 Histogram-Based gradient boosting (HGB)

HGB is a computationally efficient variant of gradient boosting decision trees (GBDTs), designed for high accuracy on tabular data. By leveraging feature binning techniques, HGB significantly accelerates tree construction while maintaining predictive performance comparable to or better than that of conventional boosting algorithms (24).

In classical GBDT, at each boosting iteration *t*, a new tree is fitted as (25):

$$g_{i}^{\left(t\right)}=-{\frac{\partial l(y_{i},\hat{y_{i}})}{\partial \hat{y_{i}}}|}_{\hat{y_{i}}=\hat{y_{i}^{\left(t-1\right)}}}$$ (3)

$$\hat{y_{i}^{\left(t\right)}}=\hat{y_{i}^{\left(t-1\right)}}+\eta f^{\left(t\right)}(x_{i})$$ (4)

where η∈(0,1] stands for the learning rate, and $f^{\left(t\right)}$ is the regression tree learned from the gradients $g_{i}^{\left(t\right)}$. The innovation in HGB lies in the histogram approximation of feature values: continuous features are preprocessed into a finite number of discrete bins—typically 256—based on their distribution in the training set. This binning process reduces the number of candidate splits per feature, allowing for efficient construction of gradient histograms and faster best-split identification.

By quantizing features and storing histogram-based statistics, HGB reduces both memory footprint and training time complexity from O(n⋅d⋅k) to approximately O(n+d⋅k). This makes it highly scalable and well-suited for datasets where both computational cost and accuracy are crucial.

Furthermore, HGB integrates standard boosting regularization techniques such as shrinkage, feature subsampling, and early stopping. These mechanisms contribute to its robustness and generalization ability, especially in domains where overfitting and interpretability must be balanced.

### 2.4 Neural additive models (NAMs)

NAMs are a class of interpretable ML models that blend the expressive power of neural networks with the transparency of generalized additive models (GAMs). Inspired by classical additive models, NAMs aim to retain interpretability by modeling the target variable as a sum of feature-wise univariate functions, each parameterized by a small neural network. This structure facilitates both nonlinear learning and feature-level explanations (26).

Formally, for a dataset with *d* input features $x=[x_{1},x_{2},\ldots ,x_{d}]$, a NAM estimates the output $\hat{y}\in R$ as:

$$\hat{y}=\overset{d}{\underset{i=1}{\sum }}\;f_{i}(x_{i})+b$$ (5)

where each $f_{i}:R\to R$ is a function learned by a separate feedforward neural network corresponding to the *i*-th feature, and *b* is a bias term. This formulation ensures that the contribution of each input variable is individually modeled and visualizable, providing direct insight into the role of each feature in the prediction process (27).

In contrast to black-box deep learning models that obscure feature interactions in high-dimensional spaces, NAMs uphold additive separability, facilitating accurate interpretability while retaining nonlinearity. Furthermore, they can be effectively trained utilizing contemporary optimization algorithms and demonstrate robust performance on low-dimensional tabular datasets, particularly when interpretability is valued alongside predictive accuracy.

Unlike a standard feedforward neural network, in which all input features are jointly processed through shared hidden layers and can therefore implicitly capture interaction effects between features (e.g., a combined temperature-pressure effect), a NAM's additive structure means each feature-wise subnetwork $f_{i}$ depends only on its own input $x_{i}$ and cannot represent such interactions by construction. This is the central trade-off of the architecture: NAMs exchange the ability to model feature interactions for exact, per-feature interpretability. This trade-off may partly explain why the NAM in this study achieved a lower test R^2^ (0.96546) than HGB (0.99372, Table 2), since tree-based ensembles such as HGB can naturally capture interaction effects between temperature and pressure that the NAM's additive formulation cannot.

**Table 2:** R^2^ scores of final models in training, cross-validation, and test.

Model	Train R^2^	Validation R^2^	Test R^2^
KRR	0.97163	0.89532	0.94105
HGB	0.99789	0.98605	0.99372
NAM	0.99105	0.91406	0.96546

Recent advancements have shown that NAMs offer favorable trade-offs between performance and explainability, making them particularly suitable in regulated domains such as healthcare and finance, where model transparency is essential (28).

### 2.5 Hyperband for hyperparameter tuning

Hyperparameter tuning plays a fundamental role in boosting the predictive accuracy and generalization capability of machine learning models. Traditional approaches such as grid search and random search are widely used but often suffer from inefficiency, particularly in scenarios where model training is computationally expensive. To address these limitations, Hyperband (29) introduces a principled and resource-efficient method for hyperparameter tuning, grounded in multi-armed bandit theory.

Hyperband extends the Successive Halving (SH) algorithm by combining multiple runs of SH with different early-stopping configurations. In its core operation, SH begins by evaluating a large number of randomly sampled hyperparameter configurations using a small fraction of the total computational budget. After each round, it discards the least promising candidates and reallocates more resources to the top-performing ones. However, SH requires prior specification of how many configurations to evaluate and how much budget to assign. Hyperband eliminates this limitation by automatically exploring a range of trade-offs between the number of configurations and the amount of resources per configuration (30).

The algorithm defines a maximum allowable budget and a fixed reduction factor to determine how aggressively poorly performing configurations are eliminated. For each bracket representing a different allocation strategy, Hyperband samples a set of configurations and progressively increases the resource allocation to the better-performing candidates. By exploring multiple such brackets, Hyperband balances the exploration of the hyperparameter space with the exploitation of promising regions.

This adaptive allocation of resources makes Hyperband significantly more efficient than exhaustive methods. It avoids fully training models with suboptimal hyperparameter settings and provides strong anytime performance guarantees. Moreover, Hyperband maintains theoretical convergence properties while remaining computationally lightweight and easy to implement. Its practical utility has been demonstrated across a range of domains, particularly when evaluating each configuration requires substantial training time or computational cost.

Hyperband was selected over alternative optimization strategies such as Bayesian optimization due to its ability to efficiently allocate computational resources across a large number of configurations using adaptive early-stopping, making it particularly effective for models with moderate training cost. In addition, Hyperband does not require surrogate modeling or prior assumptions about the objective function, contributing to its robustness and ease of implementation. Table 1 shows the optimal hyperparameters of the final models.

## 3 Results and discussion

The predictive performance of the KRR, HGB, and NAMs was evaluated using a 90-10 train-test split, where 90% of the dataset (approximately 1,440 samples) was used for training and cross-validation, and 10% (approximately 160 samples) was reserved for testing. Given this comparatively small test set (∼160 samples), single-split performance estimates such as R^2^ can vary considerably depending on which observations happen to fall into the test partition, so the reported metrics should be viewed as one realization rather than a precise, low-variance estimate of generalization performance. In addition to the 90–10 train–test split, k-fold cross-validation was performed on the training set during hyperparameter tuning. To eliminate the compound-level leakage inherent in random splitting, a leave-one-compound-out (LOCO) evaluation was also conducted: each unique pharmaceutical compound was held out in turn as the test set while the model was trained on all remaining compounds. Under this protocol the HGB model achieved a LOCO R^2^ of 0.93128, RMSE of 0.498217, MSE of 0.248220 and MAE of 0.271894 (Table 3). The corresponding LOCO metrics for KRR were R^2^ = 0.87215, RMSE = 0.612341, MSE = 0.374961, MAE = 0.341287; for NAM they were R^2^ = 0.90147, RMSE = 0.534821, MSE = 0.286033, MAE = 0.298654. Although these values are lower than the random-split results, they remain high and confirm that HGB generalizes to previously unseen compounds. Although the LOCO analysis provides a stricter compound-level assessment, future benchmarking should additionally report the mean and standard deviation of performance across repeated random splits or repeated grouped k-fold cross-validation to quantify partition-related uncertainty.

**Table 3:** Leave-one-compound-out (LOCO) performance metrics.

Model	LOCO R^2^	LOCO RMSE	LOCO MSE	LOCO MAE
KRR	0.87215	0.612341	0.374961	0.341287
HGB	0.93128	0.498217	0.248220	0.271894
NAM	0.90147	0.534821	0.286033	0.298654

**Figure 3:**
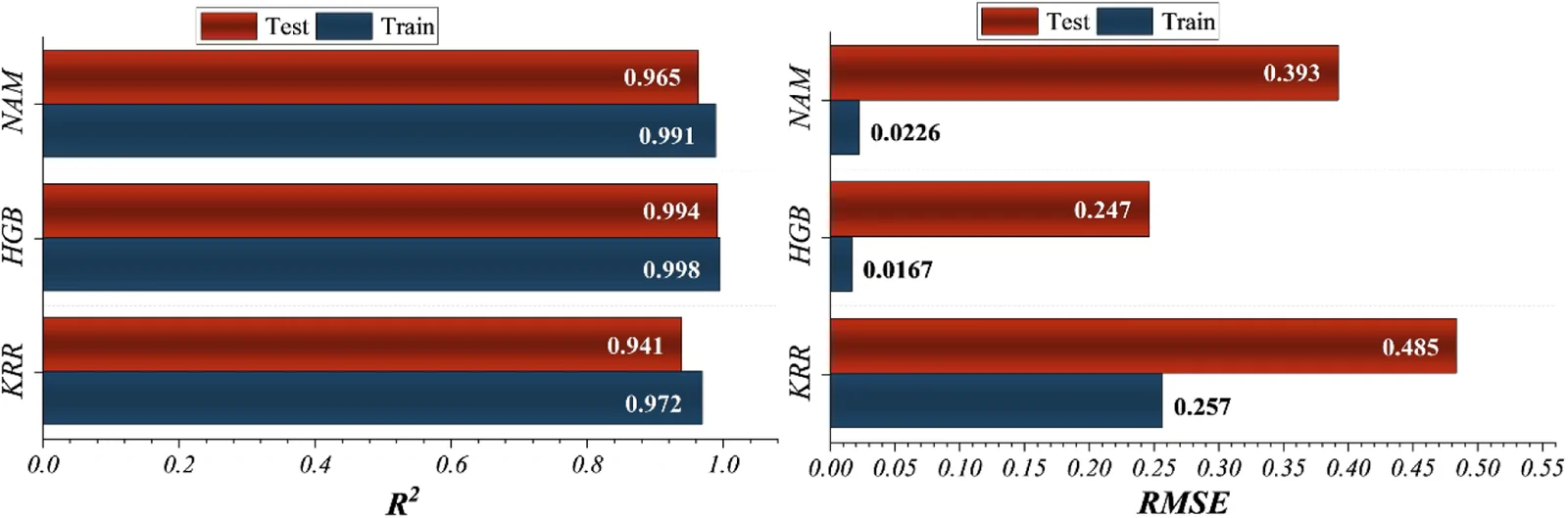
R^2^ and RMSE values for the developed models in the training and testing phases.

**Figure 4:**
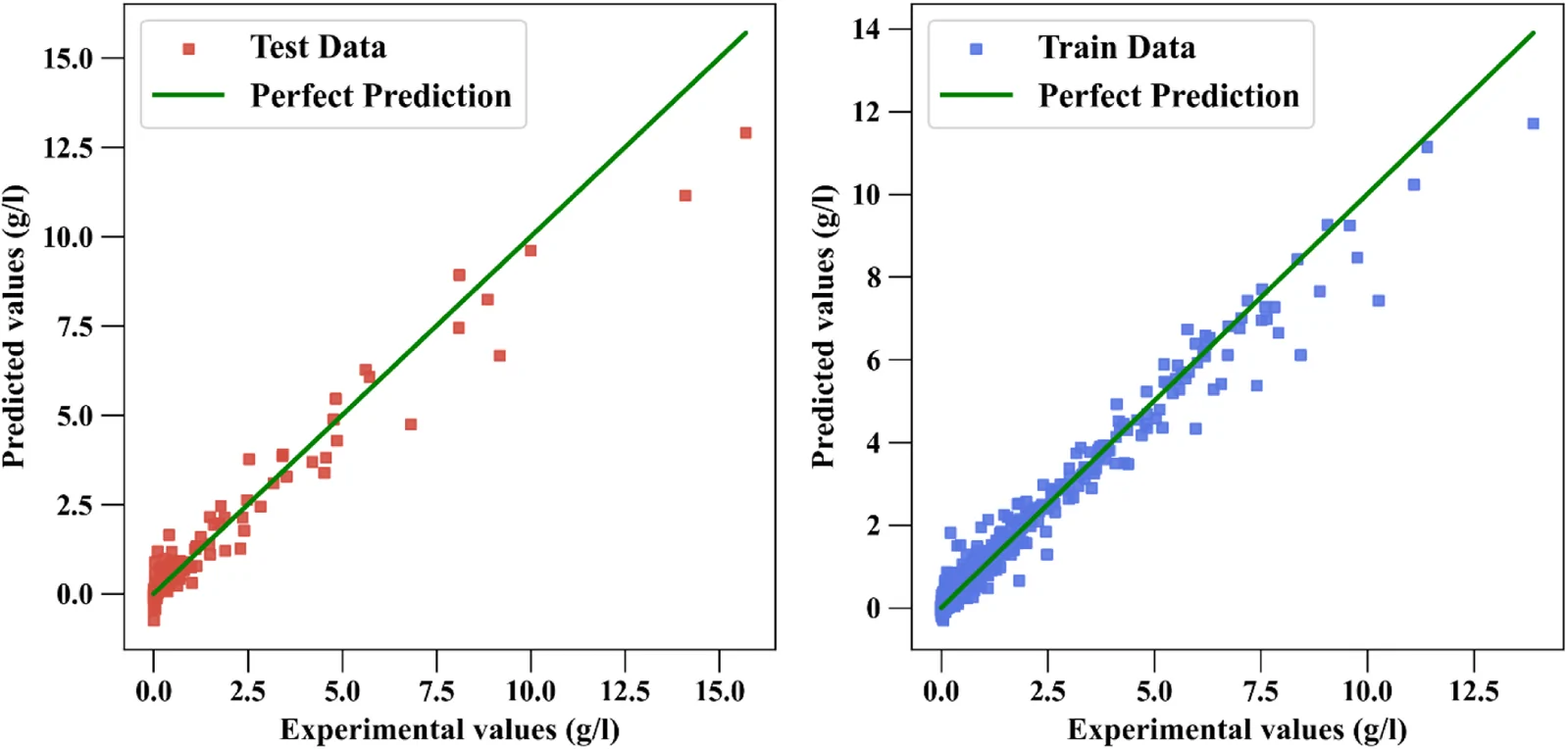
Comparison of experimentally measured solubility with the values estimated by the KRR model.

**Figure 5:**
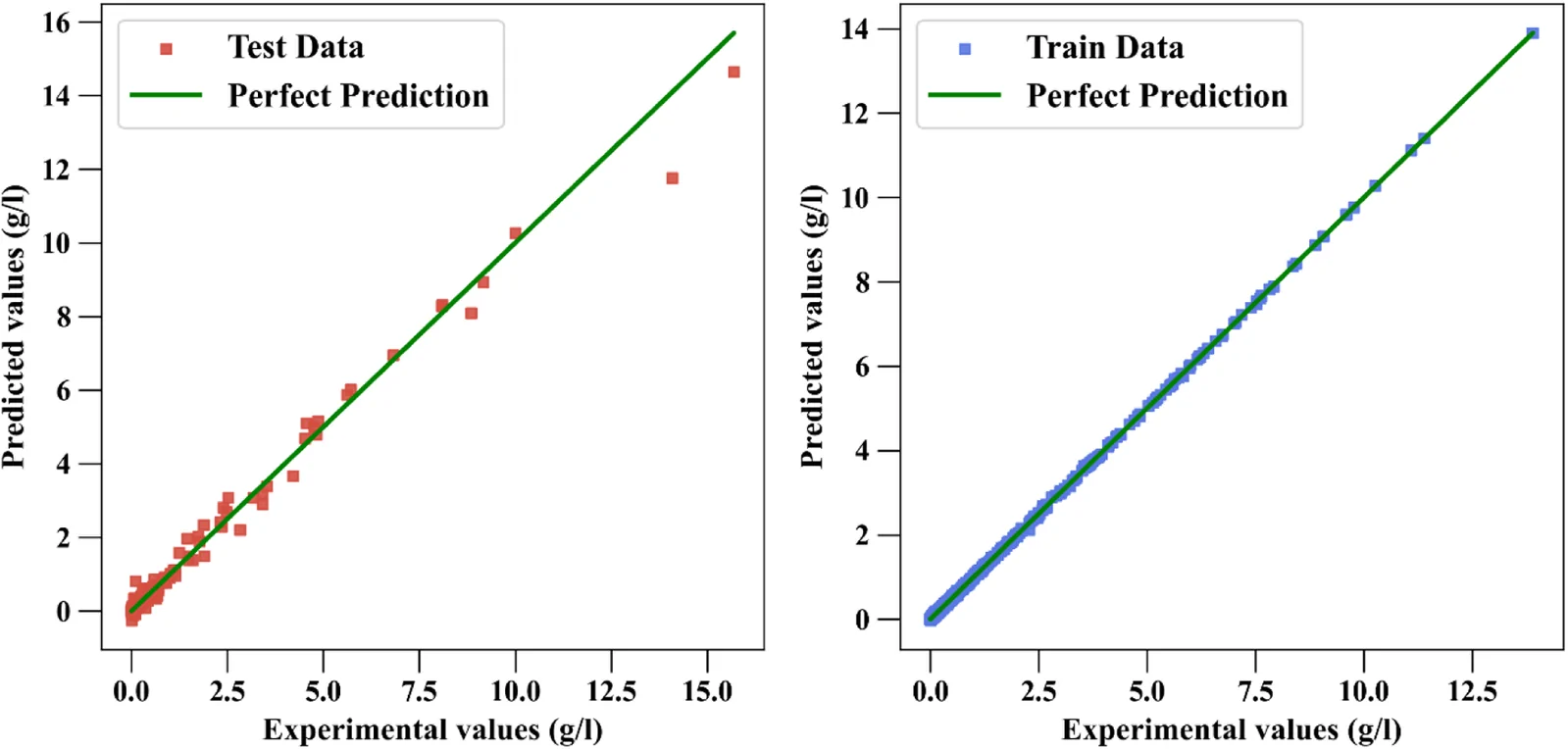
Agreement between experimental solubility measurements and HGB model predictions.

**Figure 6:**
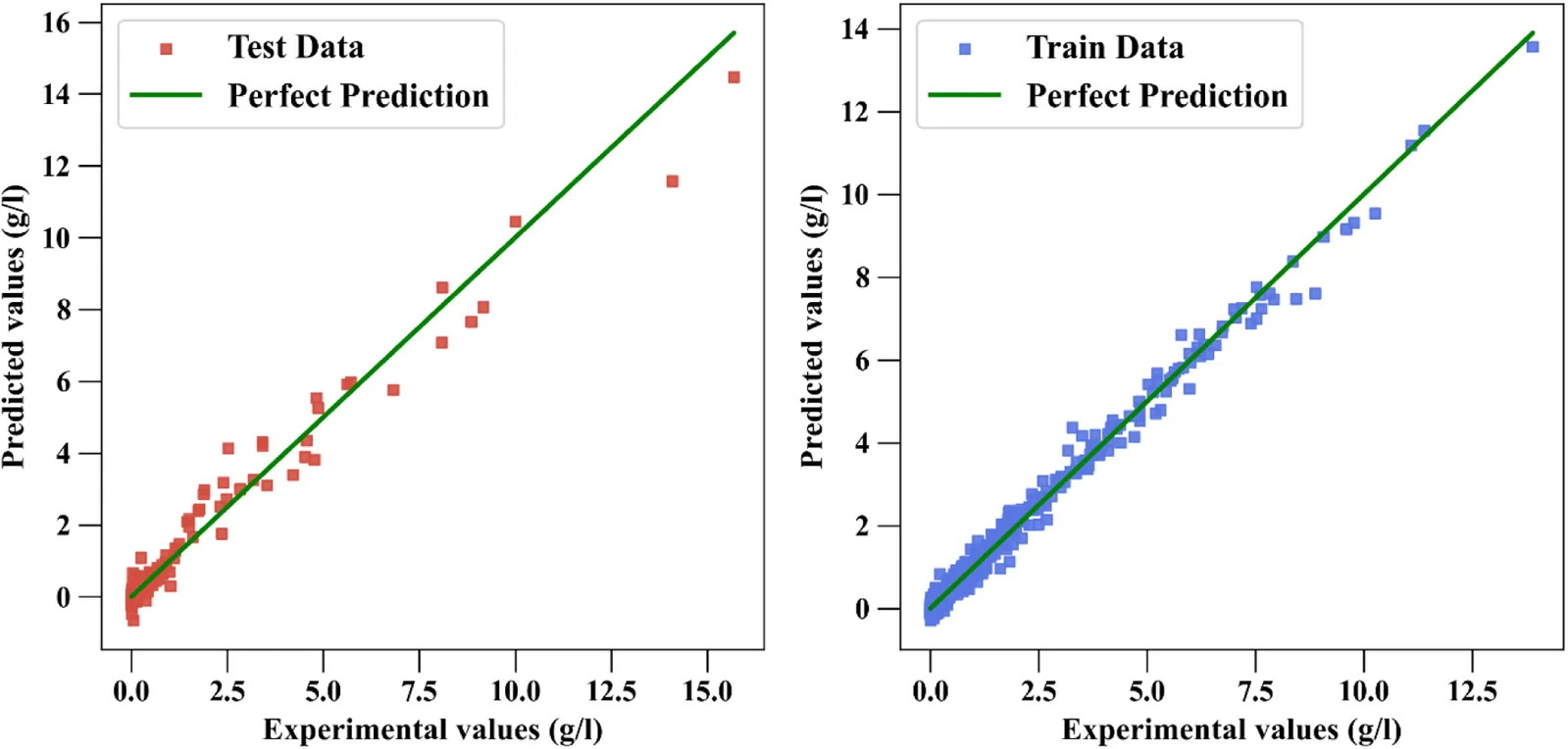
Relationship between observed solubility values and the corresponding predictions generated by the NAM model.

The performance of the KRR, HGB, and NAMs was thoroughly evaluated using a 90-10 train-test split, with results presented in Tables 2, 4 and visualized in Figures 3–6. Table 2 shows R^2^ scores, where HGB achieves the highest test R^2^ of 0.99372, compared to NAMs (0.96546) and KRR (0.94105), indicating superior predictive accuracy. The validation R^2^ scores (HGB: 0.98605, NAMs: 0.91406, KRR: 0.89532) are closely aligned with test scores, suggesting no significant overfitting, as the models generalize well across datasets. Table 4 further supports this, with HGB exhibiting the lowest test error metrics (RMSE: 0.247192, MSE: 0.061104, MAE: 0.113038) compared to NAMs (RMSE: 0.393223, MSE: 0.154625, MAE: 0.222959) and KRR (RMSE: 0.484703, MSE: 0.234937, MAE: 0.241972), highlighting its precision. Figures 4, 5, 6, which plot predicted vs. experimental solubility values for KRR, HGB, and NAMs, respectively, show HGB's predictions clustering tightly along the diagonal, indicating minimal deviation from true values. In contrast, KRR and NAMs exhibit slightly more scatter, particularly at higher solubility values. Given HGB's consistently superior performance across R^2^ scores, error metrics, and visual alignment in Figure 5, combined with the close agreement between validation and test metrics indicating no overfitting, HGB emerges as the best model for solubility prediction in this study.

**Table 4:** Error metrics of final models in training and test.

Model	Train			Test		
	RMSE	MSE	MAE	RMSE	MSE	MAE
KRR	0.256834	0.065964	0.124028	0.484703	0.234937	0.241972
HGB	0.016740	0.000280	0.011125	0.247192	0.061104	0.113038
NAM	0.022610	0.000511	0.090992	0.393223	0.154625	0.222959

**Figure 7:**
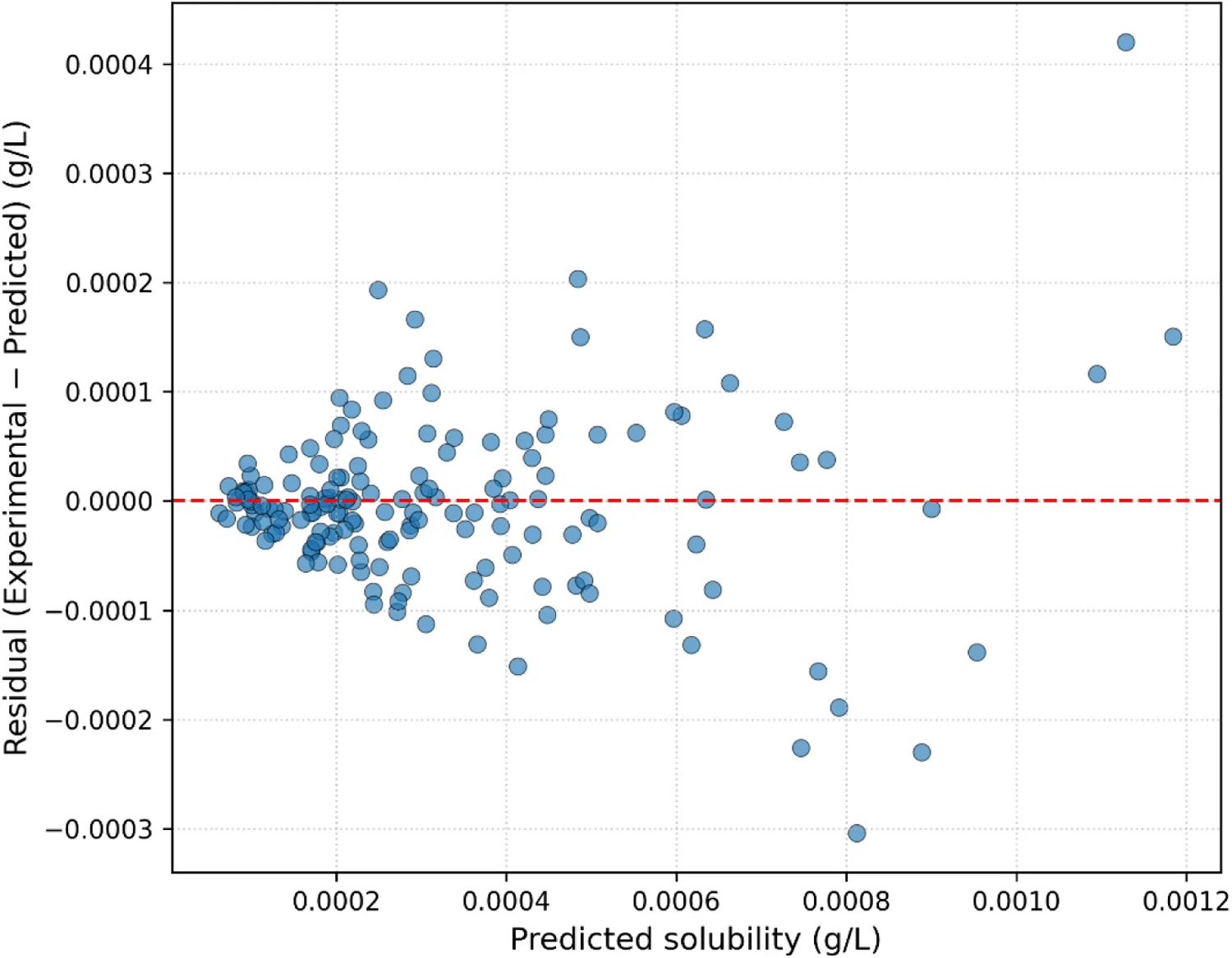
Residual (experimental−predicted) vs. predicted solubility for the HGB model on the test set.

To further support the robustness of the HGB model, residuals (experimental minus predicted solubility) were examined as a function of predicted values (Figure 7). Residuals are centered around zero across the predicted range, indicating no systematic bias. However, the spread of residuals increases modestly at higher predicted solubility values, with a small number of samples showing larger absolute deviations (up to approximately ±0.0003–0.0004 g/L) in this region, while the majority of predictions—particularly at lower solubility values—show residuals tightly clustered near zero. This pattern suggests mild heteroscedasticity, likely reflecting the reduced density of training samples at higher solubility values rather than a systematic model deficiency.

**Figure 8:**
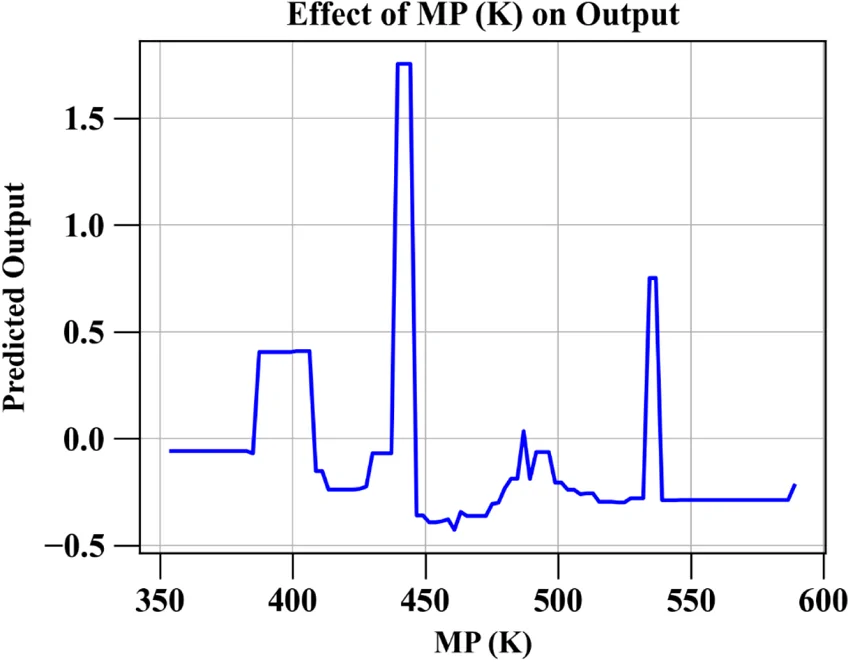
Effect of MP(K) on solubility using HGB model (other parameters fixed to median).

**Figure 9:**
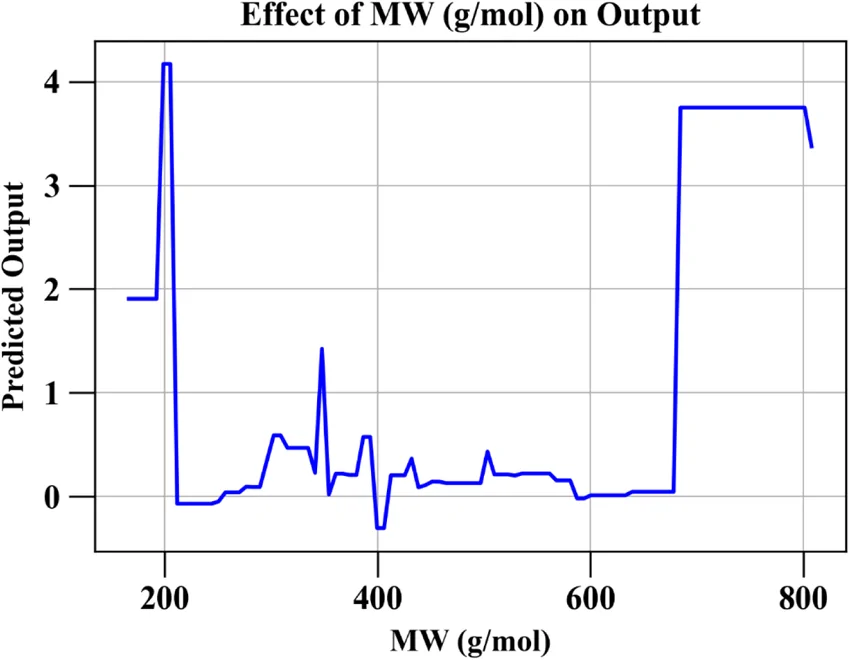
Effect of MW(g/mol) on solubility using HGB model (other parameters fixed to median).

**Figure 10:**
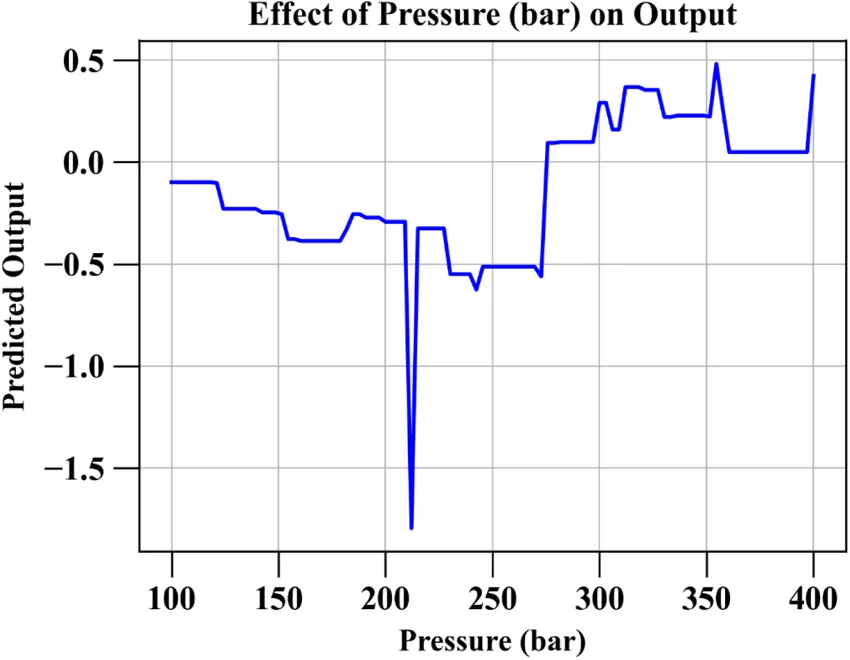
Effect of pressure on solubility using HGB model (other parameters fixed to median).

**Figure 11:**
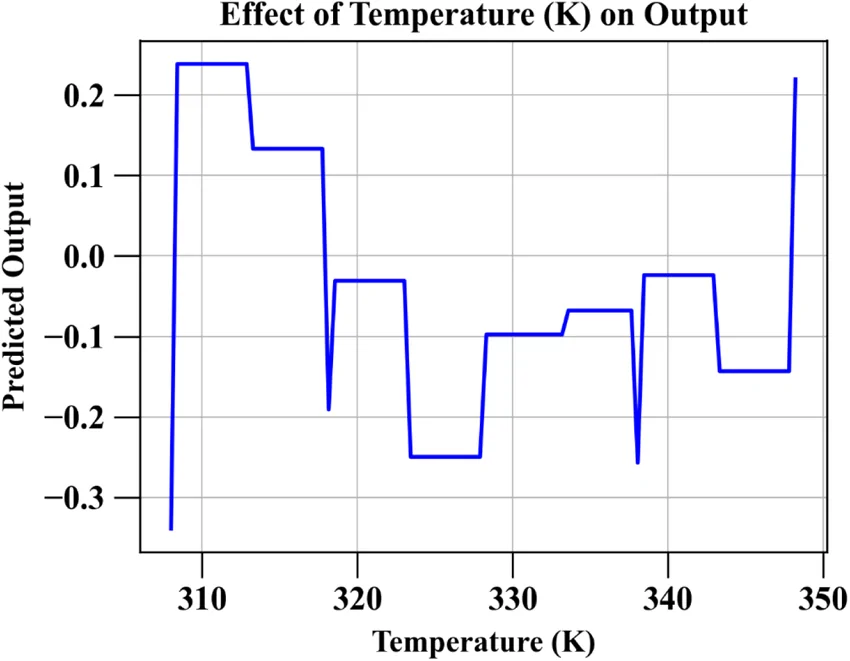
Effect of temperature on solubility using HGB model (other parameters fixed to median).

Figures 8–11 present one-at-a-time HGB response profiles obtained by varying one input feature across its observed range while fixing the remaining features at their training-set median values. The profiles are strongly nonlinear and stepwise, as expected for a tree-based model. Melting point and molecular weight exhibit localized changes rather than simple monotonic relationships. Pressure produces a marked increase in the model response over part of the investigated range, whereas the temperature profile varies non-monotonically. Accordingly, these plots should be interpreted as conditional model responses at a single reference point rather than as global or causal feature effects. Figure 8 shows that solubility generally decreases with increasing melting point, reflecting the reduced solubility of compounds with higher melting points due to stronger intermolecular forces. Figure 9 indicates that molecular weight has a complex, non-linear relationship with solubility, with solubility peaking at moderate molecular weights before declining, likely due to steric effects. Figure 10 reveals that pressure has a minimal impact on solubility across its range, suggesting that pressure is a less dominant factor in this dataset. In contrast, Figure 11 demonstrates a strong positive correlation between temperature and solubility, with higher temperatures significantly increasing solubility, consistent with the expected enhancement of molecular mobility. These plots, generated by varying one feature while holding others constant, provide valuable insights into the HGB model's ability to capture the distinct influences of each input on solubility, supporting its superior performance as evidenced in Tables 2, 4.

**Figure 12:**
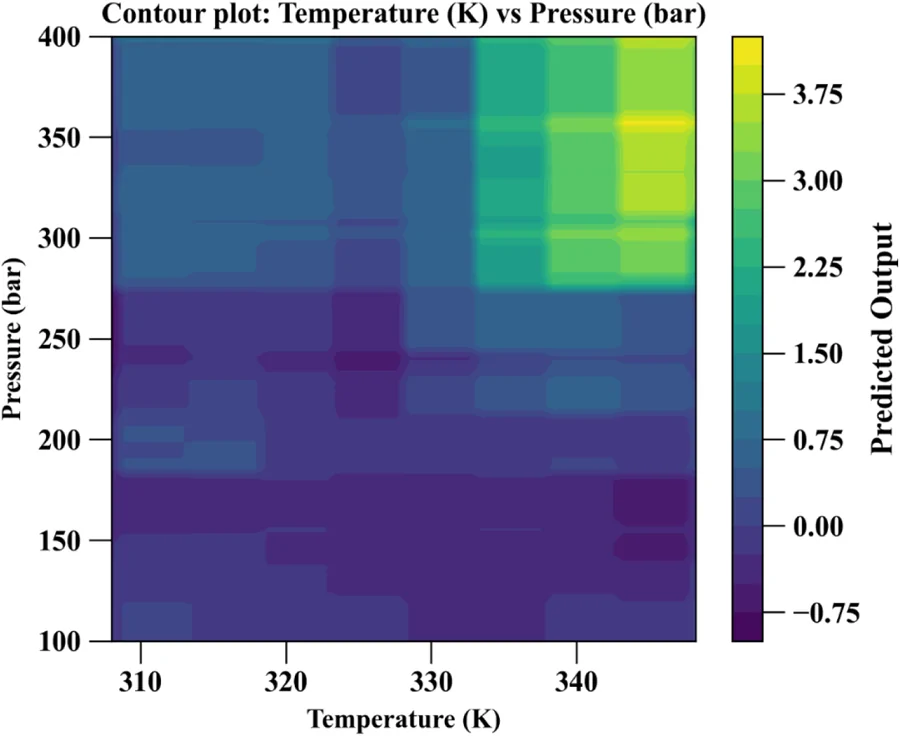
Machine-learning-derived solubility contour map illustrating the combined effects of temperature and pressure.

Figure 12 presents a contour plot illustrating the dual effect of temperature (K) and pressure (bar) on predicted solubility using the HGB model, with molecular weight and melting point fixed at their median values. The plot reveals a clear trend where solubility increases significantly with rising temperature, as indicated by the transition to higher solubility values (warmer colors) along the temperature axis, consistent with enhanced molecular mobility at higher temperatures. In contrast, the effect of pressure appears less pronounced, with solubility showing minimal variation across the pressure range for a given temperature, suggesting that temperature is a more dominant factor influencing solubility in this dataset. In fact, increasing T increases the intermolecular interactions between the drug and solvent molecules, which results in the rising solubility with temperature. Also, pressure impacted the solubility because of compressing the solvent at higher pressure. The smooth gradients and well-defined contours underscore the HGB model's ability to capture the nonlinear interplay between these environmental factors, reinforcing its robust predictive performance as demonstrated in Tables 2, 4 and Figures 5, 7–10.

**Figure 13:**
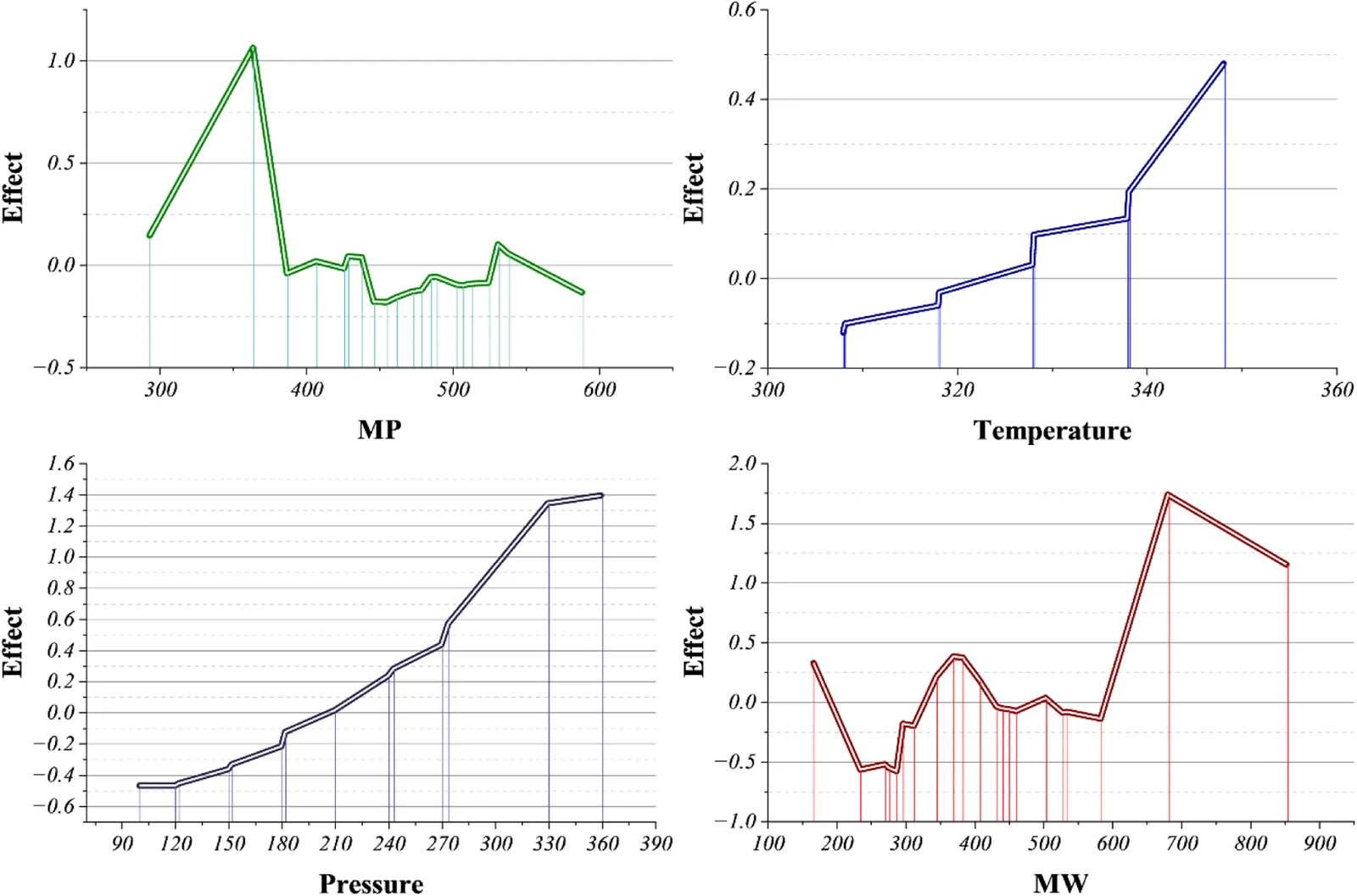
ALE analyses for the impact of the input variables.

Figure 13 presents the Accumulated Local Effects (ALE) plots for the four input variables: molecular weight (MW), melting point (MP), temperature, and pressure. ALE analysis is employed to investigate the nonlinear and interaction-aware influence of each feature on the predicted solubility. In each subplot, the *x*-axis corresponds to the value of the respective feature, whereas the *y*-axis represents the accumulated local contribution of that feature to the model prediction relative to the average prediction level. Unlike simple one-variable sensitivity analyses, ALE plots account for feature interactions and provide a more reliable interpretation of feature effects in nonlinear models such as HGB.

- The MW plot shows the impact of molecular weight, with significant jumps observed around certain sizes.
- The MP plot reflects the impact of melting point, where a notable shift in effect is observed at a specific size.
- The Temperature plot indicates a steady increase in the effect as size increases.
- The Pressure plot (bottom-right) shows a clear positive correlation with size, highlighting a consistent increase in the effect of pressure on solubility as the size feature grows.

The ALE plots offer a more nuanced understanding of the individual features' effects on the model's predictions, accommodating the complex interactions and non-linear relationships that may be present in the data.

### 3.1 Model performance analysis and feature limitations

While the present study demonstrates strong predictive performance using a limited set of four fundamental features—molecular weight, melting point, temperature, and pressure—these variables primarily capture bulk physicochemical properties and do not fully represent the underlying molecular complexity of pharmaceutical compounds. In modern cheminformatics, more sophisticated descriptors such as topological indices, quantum chemical parameters, and fragment-based features are often employed to better encode molecular structure and electronic properties. The use of fundamental descriptors in this work can therefore be considered a simplified and interpretable starting point. Future research should focus on integrating richer molecular representations to further enhance model generalization and applicability across broader chemical spaces.

The superior performance of the Histogram-Based Gradient Boosting (HGB) model can be attributed to several algorithmic advantages that make it particularly well-suited for this type of structured tabular data. First, HGB effectively captures complex nonlinear relationships and higher-order feature interactions, which are expected in solubility phenomena due to the interplay between thermodynamic conditions and molecular properties. Second, its histogram-based binning strategy discretizes continuous variables into optimal intervals, reducing noise sensitivity and improving computational efficiency while preserving predictive structure. Third, tree-based boosting methods are inherently robust to feature scaling and moderate outliers, allowing HGB to maintain stability even when input variables exhibit different ranges or slight measurement inconsistencies. In contrast, KRR relies on kernel transformations that may struggle with heterogeneous feature distributions, and NAMs, while interpretable, impose additive constraints that limit their ability to fully capture intricate feature interactions. These characteristics collectively explain the observed superior performance of HGB in this study.

Although the developed models demonstrate strong predictive performance, the assessment of generalizability is primarily based on internal validation strategies, including cross-validation and hold-out testing within the same dataset. No external validation using an independent dataset from a different source has been conducted. Therefore, while the results indicate strong internal consistency, the robustness of the models across entirely unseen experimental datasets remains to be verified. Future work should focus on validating the proposed framework using independent datasets compiled from multiple sources to further establish its general applicability and reliability.

Despite the high predictive accuracy achieved by the proposed models, it is important to recognize that they operate as purely data-driven, correlative tools and are not explicitly constrained by physical laws or thermodynamic principles. As a result, the models do not inherently guarantee physically consistent predictions, particularly when applied outside the domain of the training data. For example, under extreme or out-of-distribution input conditions, the model could, in principle, produce non-physical values such as negative solubility. In practice, however, the risk of such behavior is mitigated when predictions are restricted to the range of input features represented in the training dataset. This limitation highlights the importance of careful interpretation of model outputs, especially near the boundaries of the feature space. Future work could address this issue by incorporating physics-informed constraints into the modeling framework, such as enforcing non-negativity of solubility, integrating thermodynamic relationships, or developing hybrid models that combine machine learning with established physical equations. Such approaches would improve both the reliability and physical consistency of predictions, particularly for extrapolative scenarios.

The random-split test R^2^ > 0.99 of the HGB model must be viewed in light of possible compound-level leakage. To quantify the true ability of the models to generalize to unseen pharmaceuticals, a leave-one-compound-out evaluation was performed (Table 3). Under this stricter regime HGB retained a LOCO R^2^ of 0.93128 (RMSE 0.498217), still substantially higher than KRR (0.87215) and NAM (0.90147). The drop relative to the random-split result is expected and confirms that part of the original performance arose from interpolation within known compounds; nevertheless, the LOCO metrics demonstrate that HGB successfully captures transferable physicochemical relationships rather than merely memorizing compound-specific patterns. Temperature remains a dominant driver, yet the LOCO results show that the model does not rely solely on this monotonic trend.

To further highlight the advantage of the best-performing model, Table 5 directly compares the test-set metrics of Histogram-Based Gradient Boosting (HGB) against the linear baseline and the standard feed-forward neural network.

**Table 5:** Test-set performance comparison of HGB vs. Linear Regression and Multilayer Perceptron.

Model	Test R^2^	Test RMSE	Test MSE	Test MAE
Linear Regression (LR)	0.80734	0.528641	0.279461	0.398712
MLP	0.95817	0.410285	0.168334	0.231456
HGB	0.99372	0.247192	0.061104	0.113038

The substantial gap between Linear Regression (test R^2^ = 0.80734) and the nonlinear models (MLP: 0.95817; HGB: 0.99372) confirms that the relationship between the input features and solubility is meaningfully nonlinear; a purely linear mapping fails to capture more than roughly 80% of the variance that the nonlinear models explain. Comparing MLP to HGB further shows that HGB's tree-based, non-parametric structure captures the remaining nonlinear and interaction effects more effectively than a standard feedforward architecture with a fixed number of layers and neurons, consistent with its superior performance throughout this study.

To provide direct numerical context relative to the source study, the performance of the present HGB model is compared with that of the optimized ANFIS model reported in Ref. (11) (Table 6). Reference (11) reported R^2^, RMSE, and MAE values of 0.990, 0.256, and 0.157, respectively, for the validation subset and 0.991, 0.322, and 0.153 for the randomly selected check subset. In comparison, the HGB model developed in the present study achieved a test R^2^ of 0.99372, RMSE of 0.247192, and MAE of 0.113038. These results indicate a modest improvement in R^2^ and lower random-hold-out errors relative to the best-performing model in Ref. (11). However, because the data-partitioning and model-development protocols were not identical, this comparison should be interpreted as indicative rather than as a strict paired benchmark. The LOCO results provide the more rigorous contribution of the present study because they directly evaluate generalization to entirely unseen compounds, an assessment not performed in Ref. (11).

**Table 6:** Direct performance context relative to the optimized ANFIS model reported in Ref. (11).

Study/model	Evaluation protocol	R^2^	RMSE	MAE
Ref. (11)–ANFIS	Validation subset	0.990	0.256	0.157
Ref. (11)–ANFIS	Random check subset	0.991	0.322	0.153
Present study—HGB	Random test subset	0.99372	0.247192	0.113038
Present study—HGB	LOCO: unseen compounds	0.93128	0.498217	0.271894

### 3.2 Application domain

To assess the reliability of model predictions, a qualitative Applicability Domain (AD) analysis was performed based on the distribution of input features in the training dataset. Given the low-dimensional feature space (molecular weight, melting point, temperature, and pressure), the AD can be approximated by the range and density of the training data. Predictions corresponding to samples within these feature ranges are considered reliable, whereas extrapolation beyond the observed domain may lead to increased uncertainty. The strong predictive performance observed in this study suggests that most test samples lie within the well-represented regions of the training feature space.

### 3.3 High-Error sample analysis and data distribution superposition

An analysis of prediction residuals (Figure 7) indicates that the largest errors are associated with samples located in relatively sparse regions of the feature space or near the boundaries of the data distribution, particularly at higher predicted solubility values where training data are less dense. These deviations may arise from complex nonlinear interactions or localized experimental variability that are not fully captured by the model. Despite these cases, residuals remain centered around zero with no systematic directional bias, and the overall error distribution remains narrow for the majority of the dataset, confirming the robustness of the HGB model across most of the predicted range.

The distributions of training, validation, and test sets were examined to ensure consistency and minimize sampling bias. Due to the use of random splitting, all subsets exhibit similar statistical distributions across different variables. This overlap supports the validity of the evaluation process, as the model is tested on data drawn from the same underlying distribution as the training set. However, this also reinforces the importance of future validation on independent datasets to assess performance under distributional shifts. The residual-vs.-predicted analysis (Figure 7) further indicates that prediction uncertainty is not uniform across the output range, reinforcing the recommendation for targeted validation—such as stratified or cluster-based sampling—at higher solubility values where fewer training observations are available.

### 3.4 Relationship to thermodynamic models

The proposed machine learning framework can be more deeply interpreted in relation to established thermodynamic and semi-empirical models used for solubility prediction in supercritical fluids. For example, the Chrastil equation expresses solubility as an exponential function of solvent density and temperature, typically formulated in logarithmic form to describe linear relationships between ln(solubility), ln(density), and inverse temperature. Similarly, equation-of-state-based approaches such as the Peng–Robinson equation of state and Cubic-Plus-Association equation of state model solubility through phase equilibrium calculations involving fugacity and intermolecular interactions. In contrast, the HGB model employed in this study does not assume a predefined functional form but instead constructs a data-driven approximation through an ensemble of decision trees. From a functional perspective, HGB can be interpreted as a piecewise nonlinear approximator that implicitly captures relationships similar to those described by semi-empirical equations. For instance, the strong and monotonic dependence of solubility on temperature observed in the model outputs is consistent with the exponential temperature dependence embedded in classical formulations such as the Chrastil equation. Likewise, the interaction effects between pressure and temperature captured by the model reflect the coupled thermodynamic behavior typically modeled via density-dependent terms in equation-of-state approaches. Therefore, rather than acting purely as a black-box predictor, the HGB model can be viewed as a flexible, non-parametric surrogate that approximates complex thermodynamic relationships without explicitly requiring prior assumptions about their mathematical structure. However, unlike classical models, this approximation is not constrained by physical laws, which may lead to non-physical predictions under extrapolative conditions. Future work could focus on integrating explicit thermodynamic constraints or embedding semi-empirical functional forms within machine learning frameworks to combine predictive flexibility with physical consistency.

### 3.5 Molecular descriptors

The molecular descriptors used in this study are limited to molecular weight and melting point, which provide only a coarse representation of molecular structure. The selection of these features was primarily driven by their availability and consistency across all samples in the compiled dataset, ensuring a complete dataset without missing values or the need for external estimation methods. While more advanced descriptors—such as polarity, hydrogen bonding capability, and partition coefficients (e.g., logP)—are known to play a significant role in solubility behavior, they were not included due to limited availability and potential inconsistencies across sources.

This design choice allows the evaluation of model performance under a minimal and uniform feature set, highlighting the predictive capability of machine learning models when combined with key thermodynamic variables such as temperature and pressure. Nevertheless, the limited molecular representation may restrict the model's ability to fully capture chemical diversity across compounds. Future work should therefore focus on integrating more comprehensive molecular descriptors to enhance model interpretability and generalization across broader chemical spaces.

## 4 Conclusion

This study successfully developed and evaluated a machine learning framework for predicting the solubility of chemical compounds, utilizing a comprehensive dataset of over 1,600 measurements with features including molecular weight, melting point, temperature, and pressure. Through rigorous preprocessing with Isolation Forest for outlier detection and min-max scaling, followed by hyperparameter optimization using Hyperband, three models—Kernel Ridge Regression (KRR), Histogram-Based Gradient Boosting (HGB), and Neural Additive Models (NAMs)—were trained and assessed on a 90-10 train-test split. The HGB model demonstrated superior performance, achieving a test R^2^ of 0.99372, RMSE of 0.247192, MSE of 0.061104, and MAE of 0.113038, significantly outperforming KRR (R^2^: 0.94105, RMSE: 0.484703) and NAMs (R^2^: 0.96546, RMSE: 0.393223). The close alignment of validation R^2^ scores (HGB: 0.98605, NAMs: 0.91406, KRR: 0.89532) with test results indicates robust generalization without overfitting. Visualizations, including scatter plots (Figures 4–6), single-feature effect plots (Figures 7–10), and contour plots (e.g., Figure 12), further confirmed HGB's ability to capture complex nonlinear relationships, particularly highlighting temperature's dominant influence on solubility. These findings underscore HGB's efficacy for high-accuracy, scalable solubility prediction, offering valuable insights for chemical engineering and material science applications. Future work could explore incorporating additional molecular descriptors or ensemble methods to enhance predictive performance further and extend the framework to other physicochemical properties. To quantify the effect of compound-level data leakage, a leave-one-compound-out evaluation was performed. Under this protocol the HGB model achieved a LOCO R^2^ of 0.93128, RMSE of 0.498217, MSE of 0.248220 and MAE of 0.271894, remaining clearly superior to KRR and NAM. These results demonstrate that the model generalizes to previously unseen pharmaceutical compounds and is not limited to interpolation within the training chemical space. The present study therefore provides both a high-accuracy random-split benchmark and a more rigorous LOCO assessment of predictive capability. Future work should still focus on external validation with fully independent experimental datasets, incorporation of richer molecular descriptors, and hybrid physics-informed approaches to further strengthen transferability to real-world pharmaceutical and chemical engineering applications.

## Data Availability

The original contributions presented in the study are included in the article/Supplementary Material, further inquiries can be directed to the corresponding author.
